# Supplementary material for: Longitudinal kinetics of RBD+ antibodies in COVID-19 recovered patients over 14 months
Source: PLoS Pathog. 2022 Jun 3;18(6):e1010569. doi: 10.1371/journal.ppat.1010569 (PMC9200310; doi:10.1371/journal.ppat.1010569)
Supplement: S1 Table — A. COVID-19 recovered patients B. Follow up recovered patients (sub-cohort of the recovered patients). C. Follow up recovered patients who received one BNT162b2 mRNA-vaccine dose (recovered/vaccinated). D. Naïve vaccinees who received two doses of the BNT162b2 mRNA-vaccine. (DOCX) [file ppat.1010569.s001.docx]

| **A.** | Total cohort of COVID-19 recovered patients | Age | Max | 85 | Average | 53 |
| --- | --- | --- | --- | --- | --- | --- |
|  |  |  | Min | 20 |  |  |
|  |  | Gender | M | 97 | Total | 192 |
|  |  |  | F | 95 |  |  |
|  | F | Age | Max | 81 | Average | 53 |
|  |  |  | Min | 20 |  |  |
|  | M | Age | Max | 85 | Average | 53 |
|  |  |  | Min | 21 |  |  |

| **B.** | | | | | | Follow up cohort of COVID-19 recovered patients | Age | Max | 82 | Average | 53.2 |
| --- | --- | --- | --- | --- | --- | --- | --- | --- | --- | --- | --- |
|  |  |  |  |  |  |  |  | Min | 20 |  |  |
|  |  |  |  |  |  |  | Gender | M | 35 | Total | 66 |
|  |  |  |  |  |  |  |  | F | 31 |  |  |
|  |  |  |  |  |  | F | Age | Max | 77 | Average | 53.9 |
|  |  |  |  |  |  |  |  | Min | 20 |  |  |
|  |  |  |  |  |  | M | Age | Max | 82 | Average | 52.6 |
|  |  |  |  |  |  |  |  | Min | 26 |  |  |

| **C.** | Follow up cohort of COVID-19 recovered patients who received one dose of the BNT162b2 mRNA-vaccine | Age | Max | 77 | Average | 56.2 |
| --- | --- | --- | --- | --- | --- | --- |
|  |  |  | Min | 20 |  |  |
|  |  | Gender | M | 11 | Total | 18 |
|  |  |  | F | 7 |  |  |
|  | F | Age | Max | 77 | Average | 56.5 |
|  |  |  | Min | 20 |  |  |
|  | M | Age | Max | 76 | Average | 56 |
|  |  |  | Min | 28 |  |  |

| **D.** | | | | | | Naïve vaccinees | Age | Max | 65 | Average | 43.5 |
| --- | --- | --- | --- | --- | --- | --- | --- | --- | --- | --- | --- |
|  |  |  |  |  |  |  |  | Min | 22 |  |  |
|  |  |  |  |  |  |  | Gender | M | 4 | Total | 17 |
|  |  |  |  |  |  |  |  | F | 13 |  |  |
|  |  |  |  |  |  | F | Age | Max | 65 | Average | 43.5 |
|  |  |  |  |  |  |  |  | Min | 22 |  |  |
|  |  |  |  |  |  | M | Age | Max | 63 | Average | 46 |
