# Supplementary material for: Longitudinal kinetics of RBD+ antibodies in COVID-19 recovered patients over 14 months
Source: PLoS Pathog. 2022 Jun 3;18(6):e1010569. doi: 10.1371/journal.ppat.1010569 (PMC9200310; doi:10.1371/journal.ppat.1010569)
Supplement: S2 Table — Slopes represent the linear regression coefficients either for the fast (F) or slow (S) phases of the decay or a single slope of a linear profile. R2 and P values are indicated. The effective degrees of freedom (edf) estimated from GAMMs were used as a proxy for the degree of linearity/non-linearity relationships. An edf of 1 is equivalent to a linear relationship, an edf > 1 indicates a non-linear relationship [42]. (DOCX) [file ppat.1010569.s002.docx]

| **IgM** | **IgA** | **IgG** |  | |
| --- | --- | --- | --- | --- |
| -0.004727 | -0.00467 | -0.00457 | F.Slope | **Q4** |
| -0.002191 | -0.000342 | -0.00278 | S.Slope |  |
| 0.684 | 0.602 | 0.659 | R^2^ |  |
| <0.0001 | 0.00039 | <0.0001 | P value |  |
| 2.045 | 2.06 | 1.89 | edf |  |
| -0.003649 | -0.002561 | -0.002815 | F.Slope | **Q3** |
| 0.000693 | -0.000537 | -0.001942 | S.Slope |  |
| 0.866 | 0.593 | 0.657 | R^2^ |  |
| <0.0001 | <0.0001 | <0.0001 | P value |  |
| 2.57 | 1.97 | 1.68 | edf |  |
| -0.002036 | -0.001083 | -0.00251 | F.Slope | **Q2** |
|  |  | -0.001685 | S.Slope |  |
| 0.447 | 0.75 | 0.454 | R^2^ |  |
| 0.00124 | <0.0001 | 0.00156 | P value |  |
| 1 | 1 | 1.44 | edf |  |
| -0.00211 | -0.001022 | -0.002869 | F.Slope | **Total Cohort** |
|  |  | -0.000574 | S.Slope |  |
| 0.714 | 0.858 | 0.803 | R^2^ |  |
| <0.0001 | <0.0001 | <0.0001 | P value |  |
| 1 | 1 | 1.91 | edf |  |
