# Supplementary material for: Longitudinal kinetics of RBD+ antibodies in COVID-19 recovered patients over 14 months
Source: PLoS Pathog. 2022 Jun 3;18(6):e1010569. doi: 10.1371/journal.ppat.1010569 (PMC9200310; doi:10.1371/journal.ppat.1010569)
Supplement: S3 Table — Slopes represents the linear regression coefficients found in the first 90 DFVx2 of a profile of a linear profile. R2, and P values are indicated. The effective degrees of freedom (edf) estimated from generalized additive mixed models were used as a proxy for the degree of linearity/non-linearity relationships. An edf of 1 is equivalent to a linear relationship, an edf > 1 indicates a non-linear relationship [42]. (DOCX) [file ppat.1010569.s003.docx]

|  | **IgG** | **IgA** | **IgM** |
| --- | --- | --- | --- |
| Slope | -0.0126 | -0.0139 | -0.0081 |
| R^2^ | 0.922 | 0.857 | 0.773 |
| P value | <0.0001 | <0.0001 | <0.0001 |
| edf | 2.123 | 2.852 | 2.421 |
