# Supplementary material for: Longitudinal kinetics of RBD+ antibodies in COVID-19 recovered patients over 14 months
Source: PLoS Pathog. 2022 Jun 3;18(6):e1010569. doi: 10.1371/journal.ppat.1010569 (PMC9200310; doi:10.1371/journal.ppat.1010569)
Supplement: Attachment — Submitted filename: PLoS Pathogens D-21-02063 GCI.pdf [file ppat.1010569.s011.pdf]

The key aim of the manuscript from Eyran and colleagues is to describe the longitudinal kinetics of RBD+ antibodies in recovered COVID-19 patients over the course of 14 months, with or without two-dose vaccination with Pfizer/BioNTech BNT162b2, and how this compares to antibody persistence in n=17 highest titer (Q4) naïve vaccinees. In broad outline, the authors present data in four figures to establish the following conclusions:

° Their assay for the measurement of antibody levels exhibits high reproducibility.
° RBD-directed antibody levels within the cohort differ between males and females for IgG and IgA; higher levels are associated with disease severity; and antibody levels decay over the course of 14 months.
° The rate of temporal antibody decay can be associated with the "robustness" (titer) of the early response (i.e., the decay rate is significantly faster for those patients in whom the antibody levels at the earliest time point (visit 1 "V1") ranked in the highest quartile (Q4).
° Antibody levels in recovered patients decay more slowly compared to naïve vaccinees.

Much of the data presented in the manuscript agrees with prior published reports and is not novel; however, the computationally defined observation that antibody decay rates may differ between naturally infected vs. vaccinated individuals is potentially novel.

<u>Major Concerns</u>:

I have no requests for any substantial revisions; the manuscript is well-written.

The fact that only RBD-directed antibodies were studied should be clarified in the Title.

The fact that the differences in antibody kinetics between recovered COVID-19 patients and naïve vaccinees (n=17) was analyzed only for the highest quartile (Q4 highest titer) at first visit (V1) recovered patients — this deserves further clarification in the Discussion.

Given the observation that antibody decay rates in recovered patients correlate with quartile titer at V1, such a quartile-dependency in decay rate may be true for naïve vaccinees, too. In this regard, the analysis by Eyran *et al.* could be enhanced by the inclusion of more naïve vaccinees, especially those whose vaccine-elicited titers reach only Q3, Q2 or Q1. (I am not asking for additional data [unless it happens to exist] but feel that this limitation of the study deserves more airplay in the Discussion.)

The fact that this study lacks analysis of antibody responses directed to the spike ectodomain, and has focused exclusively on the RBD, should be discussed. For instance, the antibody decay rate of S-directed titers between recovered patients vs naïve vaccinees might be statistically indistinguishable, thus diminishing the key observation reported in the current manuscript (namely, a difference in antibody persistence of RBD-directed IgG between these two groups).

Minor Concerns:

There is no callout of Fig. 3c in the caption.

It is unclear how exactly the statistical tests were applied in Fig. 3c

The first paragraph of the Discussion is dispensable and could be deleted.
