# Supplementary material for: Longitudinal kinetics of RBD+ antibodies in COVID-19 recovered patients over 14 months
Source: PLoS Pathog. 2022 Jun 3;18(6):e1010569. doi: 10.1371/journal.ppat.1010569 (PMC9200310; doi:10.1371/journal.ppat.1010569)
Supplement: Attachment — Submitted filename: Point by Point final.pdf [file ppat.1010569.s012.pdf]

Friday, January 21, 2022

Dear PLOS Pathogens editors,

We would like to begin by thanking you and the reviewers for the helpful, productive, and constructive reviews and comments for our manuscript entitled; “**Longitudinal kinetics of RBD$^{+}$ antibodies in COVID-19 recovered patients over 14 months**” manuscript number, PPATHOGENS-D-21-02063. As a result of this review process, we believe our manuscript to be much improved.

We have carefully considered and addressed each of the reviewers’ comments and revised the manuscript according to their suggestions.
We have also complied with the editorial requests to style the manuscript according to *PLOS pathogens* formatting guidelines.

Detailed point-by-point responses to each of the reviewers’ comments can be found below.

We thank you for the consideration of our work and look forward to hearing from you.

Sincerely,

Yariv Wine, PhD

## Reviewer #1

## Part I

**Reviewer's comment:** "In this paper, Eyran and colleagues investigated the RBD-specific antibody kinetics in a cohort of 200 recovered patients (some of which were also vaccinated). They found that RBD-specific antibody levels decreased faster for those antibodies that were associated with the robustness of the response thus, recovered patients that exhibit elevated antibody levels at the first visit, experience faster decay. The authors also studied the longitudinal kinetics differences between recovered -patients and naïve BNT162b2 vaccinees and found a significantly faster decay in naïve vaccinees compared to recovered patients."

**Response:** We thank the reviewer for the careful reading and accurate understanding the content of the paper in detail.

**Reviewer's comment:** "This study investigates on a relatively large-scale vaccine dynamics over time and is therefore of interest in shaping health decisions regarding booster vaccinations."

**Response:** We thank the reviewer for acknowledging the importance of the manuscript in facilitating the shaping of health decisions regarding COIVD-19 vaccination. Indeed, as the reviewer accurately describes, our successful collaboration with HaSharon hospital - Rabin Medical Center, enabled the establishment of a relatively large cohort while also providing real-time results to both officials and the individuals participating in the study. This data was further disseminated to the relevant government bodies, thus facilitating the decision making with regard to the third vaccination in Israel.

**Reviewer's comment:** "However, data presentation, data interpretation, and embedding in existing literature need to be improved."

**Response:** We have taken the reviewer's comments regarding improvement of data presentation and interpretation and citing of existing literature into serious consideration and have revised the manuscript accordingly. We have improved the graphical representations and accompanying descriptive texts and have made a genuine effort to improve citations of existing literature. We have addressed each of the reviewer's comments specifically (elaborated in part II of the reviewer's report and presented below) thus, allowing us to address all comments in a focused and detailed manner. We thank the reviewer for the constructive comments, as they have helped to substantially improve the manuscript.

## Part II

**Reviewer's comment:** "I feel like it's not super clear to what the current work differs from previous work. Can the authors explain this more extensively in intro and discussion to sufficiently differentiate themselves from previous work – this would be especially important for those scientists who are not covid experts (like myself)"

**Response:** We thank the reviewer for this comment regarding the need to highlight how this study is different from previous reported studies. Indeed, several groups have reported the temporal dynamics and/or kinetics of SARS-CoV-2 specific antibodies following infection and

to a lesser extent following vaccination. These reports are important as they informed the community on the duration of immunity and the predicted protection. We have highlighted these reports in the introduction section, along with underlining the methodologies used to determine the longitudinal kinetics of the antibody response. However, ***there are still knowledge gaps*** with regard to the longitudinal kinetics of SARS-CoV-2 specific antibodies following recovery, including: i) duration following onset of symptoms (previous reports analyzed the kinetics in a relatively short period following the onset of symptoms); ii) lack of stratification by the robustness of the response (previous reports did not take into account the heterogenicity of the antibody response among COVID-19 recovered patients when analyzing the antibody kinetics).

Our study was conducted on a cohort of COVID-19 recovered patients with follow up samples across a maximum of 14 months following the onset of symptoms. Moreover, we applied a new approach to investigate the longitudinal kinetics by stratifying patients to groups according to their antibody levels at the first visit. This stratification approach allowed us to examine whether the antibody decay rate is associated with the robustness of the antibody response. Overall, our study provides new insights into the persistence of antibodies at the level of individuals who exhibit similar antibody response characteristics and their longitudinal kinetics over a relatively long period of time following the onset of symptoms.

**Reviewer's comment:** [Bottom of page 11]
"As recovered patients received a vaccine at various DFS, we extrapolated the antibody levels to the day that the patients received the vaccine using the regression formula. The antibody levels at 8 DFV were extrapolated as well by using the regression formulas obtained from naïve vaccinees (Figure 4e)"
Is this really valid? If yes, I'm not sure I follow. Antibody levels at 8 DFV can only be predicted like that if the regression is the same in recovered vaccinees and naive vaccinees, right? Why is there no regression analysis of recovered vaccinees? Please explain.

**Response:** We thank the reviewer for the request for a clearer and understandable explanation to our approach. We have addressed this question in the revised manuscript – see Results section. To support the understanding of the approach, we have provided an additional supplementary Figure (S5 Fig), and the revised version includes the explanation in lines: 248-277

With regard to the reviewer's comment – "Why is there no regression analysis of recovered vaccinees": We did not have more than one time point following vaccine for the recovered/vaccinated individuals, and for this reason we applied the regression model obtained from the available time points prior the vaccination in order to extrapolate the additional data points (i.e. 8 DFV).

**Reviewer's comment:** "Fig. 3b and c in the IgG column no real difference in regression discernible by eye yet $p<0.0001$ due to log scale? Please discuss. Also, add data points in Fig 3c. And change red/green to a colorblind safe color scheme."

**Response:** We thank the reviewer for this important comment. Indeed, a graphical error was made in Fig. 3c, resulting in a graph where no real difference in regression was discernible to the naked eye. We have now corrected the graphical error, as is shown in new Fig 4B and Fig 5 (Fig 3b is now Fig 4B and Fig 3c is now Fig 5). Of note, based on the new model applied

(GAMM) the regression coefficients have changed and some of the coefficients were determined by the linear regression of the two-phase decay model (Fast and Slow).

## Part III

**Reviewer's comment:** "Low resolution of most figures, please improve figure resolution."

**Response:** We apologize for the low quality of the Figures. It seems that while converting the manuscript to pdf format, the figure resolution was reduced substantially. As proposed by PloS Pathogens editorial team, we have used the PACE system to ensure that the high resolution of the manuscript figures is preserved.

**Reviewer's comment:** Fig 1:
"a: Figure does not include the vaccine patients (and depiction of DFV time points)"

**Response:** We thank the reviewer for pointing out the absence of the naïve vaccinee cohort in the new Fig 1. We have revised the Figure and now it includes a description of both cohorts.

**Reviewer's comment:** Fig 1:
b: gender → sex (change also in the text)
B: explain MI/SE in figure or just spell it out
B: the legend inside the age circle excludes the ages 51 and 51. Please correct numbers and sign. Same, in the text on page 7, you write <50 and >50, which excludes 50.

**Response:** The reviewer's comments on these figures is much appreciated and the figures have been revised and explained in the new version of the manuscript.
b: gender was changed to sex (also in the text)
B: MI/SE abbreviation in the figure was explained in the figure legend. Mild - Mild; Sev - Severe
B: We have revised the figure so the age cutoff is 60. Thus, one group will include all the ages below or equal to 60 and one group all ages higher than 60 (excluding 60). We have corrected number and signs and the text on page 7: *≤60 and >60.*

**Reviewer's comment:** Fig 1:
C: is a corr coefficient of 0.4 really signaling high reproducibility? Please discuss. And can you also discuss how this variation may influence the variability of results obtained in the later figures?

**Response:** We agree with the reviewer's comment regarding the correlation coefficient. We re-visited the correlation test that was applied for these data and considered the Pearson correlation test to be a better fit for this analysis. The results for this test were in accordance with the plots and show the correlation coefficients of r=0.8574, 0.6591 and 0.7805 for IgG, IgA, and IgM respectively. Correlation is an effect size and so we can describe the strength of the correlation using the guide that Evans (Evans, J. D. Straightforward Statistics for the Behavioral Sciences. Brooks/Cole Publishing Company, 1996) suggests for the absolute value of r as follows:
: 00-.19, "very weak"; .20-.39, "weak"; .40-.59, "moderate"; .60-.79, "strong"; .80-1.0, "very strong". Thus, we have revised the description of the correlation results that can be found in lines 140-143.

Hence, the moderate to strong positive correlation, together with of the P value for the significance test of the correlation coefficient results, suggest that the measured data can be used as a reliable source for the calculation of the longitudinal kinetics.

**Reviewer's comment:** Fig 2:
"Is it possible to add units to y-axes?
Did you consider multiple-testing correction for your analyses?"

**Response:** As suggested, y-axis units have been added. With regard to multiple-testing correction for our analysis, the data source that the graph was based on is derived from serum samples that were collected mostly from individuals at one time point; hence, most of the samples are not repeated measurements. To strengthen the statistical power of the analysis, we plotted both repeated measurement data points as well as single measurement data points (which are the majority of the data points). Thus, we decided to apply multiple testing for this analysis as suggested by the reviewer. The data now is analyzed using the generalized additive mixed model (GAMM), which includes the samples that have repeated measures.

**Reviewer's comment:** Fig 2.c note:
C: can you show the data points?

**Response:** In Fig 3D (previously Figure 2c) we show a bar plot for a simple visual representation of the comparison of the regression coefficients obtained for IgG, IgA, and IgM. In practice, this comparison was carried out by applying a comparison of fits between the resulting regressions (linear or two-phase decay) from the GAMM analysis performed on the cohort and shown in Figure 3A-C. Thus, all the data points (representing antibody levels for each isotype) were plotted in a single graph for the purpose of calculating the multiple "comparison of fits" of the slope obtained for each isotype. Since the graph derived from this comparison is overloaded and visually incomprehensible, we chose to simplify it via the presented bar plot which demonstrates the significant differences between slopes for each isotype obtained by multiple comparison. Nevertheless, the individual data point can be seen in S1 Fig and the "clean" version of the graph remains in the main text (Figure 3D). We hope the reviewer finds this explanation satisfactory.

**Reviewer's comment:** "Is 17 individuals in the naive vaccinated and 20 in recovered+vaccinated cohort enough? Please discuss."

**Response:** We thank the reviewer for this important observation. Unfortunately, under the ethical restrictions, our only accesses to naïve vaccinated individuals was from the institute's workers who agreed to volunteer to participate in this study. For this reason, our resources were limited, and thus we reached a pool of only 17 individuals. Nonetheless, it is important to note that this cohort was used as a means for a relative comparison and understanding of the recovered cohort and not as the main studied group. We have added an expanded clarification on this point in the revised manuscript, so that the role this cohort in the study will be clear to readers from all disciplines. This revision can be found in the added "Limitations of study", lines 366-372.
Nevertheless, the fact that the cohort of naïve vaccinees included many repeat sample points enabled us to enhance the statistical power of the analysis by using a mixed model regression analysis (GAMM).

**Reviewer's comment:** [Confusingly worded bottom of page 11]
"Based on the calculated temporal kinetics we found that approximately 80% of recovered vaccinees experienced an increase in IgG levels reaching the highest quartile (Q4) as opposed to 50% of them being designated to Q4 following recovery (Figure 4f)"

**Response:** We thank the reviewer for the request for a more understandable explanation of the matter. Now, we have provided a better description of the presented results in the revised manuscript, so that it may be clearer to readers from all disciplines. The revised description can be found in the new version of the submitted manuscript in the "Results" section, lines 267-271.

**Reviewer's comment:** "Would like to know: What about different vaccination intervals (e.g., 6 weeks instead of 3 in between shots). What about 2*AstraZeneca and AstraZeneca + mRNA booster and single-shot vaccines?"

**Response:** We thank the reviewer for this very important question. Indeed, the effects of a different vaccination interval of BNT162b2, vaccination with 2*AstraZeneca vaccines, AstraZeneca + mRNA booster and single-shot vaccines should all be examined over time. However, for ethical reasons, we were not able to request, instruct or encourage donors to receive vaccination in any manner, timing, or frequency, etc. The consistency in the vaccination profile of our cohort derived from the Israel Ministry of Health's vaccination campaign which presented clear and uniform guidelines for Israeli residents. In addition, at the time of this study, vaccination in Israel was only via the Pfizer BNT162b2 mRNA vaccine. The only additional vaccine available was Moderna, which only arrived in Israel in July 2021. Therefore, no alternative vaccine source was available for us to examine at the time of our study.

The different intervals between vaccine doses is a very interesting and important point. For example, the regimen that was used in UK gave better results with regard to the longevity of immunity. The vaccination regimen in Israel was strictly applied in accordance with Pfizer's recommendation, i.e., a three-week interval between doses, thus we could not obtain samples to support the study of this interesting research question.

---

## Reviewer #2:

## Part I

**Reviewer's comment:** "The manuscript by Dr. Eyran et al. describes the longitudinal kinetics of SARS-CoV-2 antibodies post-infection and vaccination. The manuscript has interesting data and would benefit from additional grammatical editing as there are numerous errors throughout. There are also major concerns with the statistical methods, loss-to-follow up and small sample sizes."

**Response:** We thank the reviewer for carefully reading, accurately understanding and summarizing the manuscript, and for acknowledging that the report presents interesting data. We have revised the manuscript according to the reviewer's comments while addressing the concerns regarding the errors throughout the manuscript, statistical methods and loss-of-follow up and small sample sizes.

## Part II

**Reviewer's comment:** “The nonlinear regression models in table 2 do not appear to fit the data particularly well. It looks as though there is a fairly rapid decline that then levels out, but the models do not reflect that. I suggest trying a Gaussian generalized additive mixed model. This model could then also be used to look at decay based on starting antibody quartiles."

**Response:** We thank the reviewer for this important comment. Indeed, the regression model that we used does not fit the data well when applied on the whole dataset (including single visit and multiple visit patients). This result is due to the high heterogenicity of the antibody levels of the recovered patients. In fact, this was our motivation in establishing an alternative strategy to evaluate the antibody decay rate. This strategy is described in the text and summarized in the new Figure S3. The stratification strategy enabled us to calculate the decay rate in a group of patients who exhibited similar antibody levels at the first visit (V1).

As suggested by the reviewer, we applied the generalized additive mixed model (GAMM) to evaluate the decay in Fig 3A-C and Fig 4B. For Figure 3A-C the model was better fitted to the data points. We thank the reviewer for highlighting this problem, as it helped to substantially improve the manuscript.

**Reviewer's comment:** “There was substantial loss to follow-up over the 4 time points in the post-infected individuals with only 12.2% presenting for the 4th draw. It is highly likely that these individuals are not representative of the general population."

**Response:** We thank the reviewer for this very important observation. Indeed, we have added in the revised manuscript a clarification in the "limitations of this study" section, with regard to donor compliance for follow-up sampling, and therefore the limited certainty of the results and conclusions. The clarification of this can be found in the added "Limitations of study" sub-section, line 364-367.

**Reviewer's comment:** "At a minimum basic information on the characteristics (age, sex, severity) of individuals that contributed at each time point should be presented along with test statistics to evaluate differences."

**Response:** The reviewer's request for an addition of presented information on the characteristics (age, sex, severity) of individuals who contributed at each time point is very much understandable. However, we found this kind of presentation sub-optimal and disproportional to the facts. This is the sole reason that while aiming for a 3-month interval between blood sampling, in practice, we had no executive control of the matter (participants would arrive on their own accord). This results in a non-uniform sampling time frame between participants.

**Reviewer's comment:** "Further, then stratifying by baseline antibody levels leaves very small sample sizes at the later timepoints."

**Response:** While we acknowledge the reviewers very valid point that stratifying the cohort leads to smaller groups, subsequently leading to small sample sizes at the later timepoints, we still stand behind this chosen strategy as it is a main pillar of this study's novelty. This strategy allowed us to systematically compartmentalize the antibody measurements in a relatively simple scaling system (Q1-Q4), a method that has not been used previously. The method is not

only novel for this field as a means of serological classification, but it also proved a sufficient and ethical means of incentive for repeat sample donation.

**Reviewer's comment:** "The vaccine cohort is extremely small, with only 17 individuals, limiting it's usefulness."

**Response:** We thank the reviewer for this important observation. Unfortunately, under the ethical restrictions, our only accesses to naïve vaccinated individuals was from the institute's workers who agreed to volunteer to participate in this study. For this reason, our resources were limited and thus we reached a pool of only 17 individuals. Nonetheless, it is important to note that this cohort exists as a means for a relative comparison and understanding of the recovered cohort and not as the main studied group. We have added an expanded clarification on this point in the revised manuscript, so that the role this cohort in the study might be clear to readers from all disciplines. This revision can be found in the added "Limitations of study" sub-section, line 367-373.
Nevertheless, as the cohort of naïve vaccinees included many repeat sample points, we were able to enhance the statistical power of the analysis by using a mixed model regression analysis (GAMM).

**Reviewer's comment:** "It is not clear how the antibody levels were "extrapolated" to day 0 and day 8. Given the small sample size, it would seem to be very error prone."

**Response:** We thank the reviewer for the request for a clearer and understandable explanation to our approach. We have addressed this matter in the revised manuscript- see the Results section. To support the understanding of the approach, we have provided an additional supplementary Figure (S5 Fig) and the revised manuscript includes explanation in lines 248-277.

With regards to the small sample size - Indeed, we have added in the revised manuscript a clarification in the "limitations of this study" section, with regard to donor compliance for follow-up sampling, and therefore the limited certainty of the results and conclusions. The clarification of this can be found in the added "Limitations of study" sub-section, line 364-367.

## Part III

**Reviewer's comment:** "Results are presented in the introduction."

**Response:** We thank the reviewer for this important note. We have edited the Introduction accordingly in the revised manuscript.

**Reviewer's comment:** "Some of the figures are hard to see."

**Response:** We thank the reviewer for this important observation and apologize for the inconvenience. We have performed, to the best of our ability, optimization of all attached figures in the revised manuscript and have also used the PACE image converter, as recommended by the PLOS Pathogens editor.

---

## Reviewer #3:

## Part I

**Reviewer's comment:** “The key aim of the manuscript from Eyran and colleagues is to describe the longitudinal kinetics of RBD+ antibodies in recovered COVID-19 patients over the course of 14 months, with or without two-dose vaccination with Pfizer/BioNTech BNT162b2, and how this compares to antibody persistence in n=17 highest titer (Q4) naïve vaccinees. In broad outline, the authors present data in four figures to establish the following conclusions:"

° Their assay for the measurement of antibody levels exhibits high reproducibility.

° RBD-directed antibody levels within the cohort differ between males and females for IgG and IgA; higher levels are associated with disease severity; and antibody levels decay over the course of 14 months.

° The rate of temporal antibody decay can be associated with the “robustness” (titer) of the early response (i.e., the decay rate is significantly faster for those patients in whom the antibody levels at the earliest time point (visit 1 “V1”) ranked in the highest quartile (Q4).

° Antibody levels in recovered patients decay more slowly compared to naïve vaccinees.

**Response:** We thank the reviewer for the thoroughly reading, accurately dissecting and eloquently summarizing the content of the paper.

**Reviewer's comment:** "Much of the data presented in the manuscript agrees with prior published manuscripts and is not novel; however, the computationally defined observation that antibody decay rates may differ between naturally infected vs. vaccinated individuals is potentially novel."

**Response:** Indeed, we approached this study's main objective consciously aware that the desired result is not novel in nature but rather that the novelty lies in the approach taken to achieve the results. In addition, our extensive literature survey indicated that our 14+ month, longitudinal, and data stratification (Q1-Q4) analysis is in fact novel.

## Part II

**Reviewer's comment:** “I have no requests for any substantial revisions; the manuscript is well-written."

**Response**: We thank the reviewer for their encouraging remark, in addition to their previous practical notes.

**Reviewer's comment:** “The fact that only RBD-directed antibodies were studied should be clarified in the Title."

**Response:** We thank the reviewer for this important remark. We have changed the Title accordingly.

**Reviewer's comment:** “The fact that the differences in antibody kinetics between recovered COVID-19 patients and naïve vaccinees (n=17) was analyzed only for the highest quartile (Q4 highest titer) at first visit (V1) recovered patients — this deserves further clarification in the Discussion."

**Response:** We thank the reviewer for the request for this important clarification. We have provided an expanded clarification on this point in the revised manuscript, so that the reason for this particular comparison might be clear to readers from all disciplines. This expansion can be found in the "Discussion", line 328-338.

**Reviewer's comment:** “Given the observation that antibody decay rates in recovered patients correlate with quartile titer at V1, such a quartile-dependency in decay rate may be true for naïve vaccinees, too. In this regard, the analysis by Eyran et al. could be enhanced by the inclusion of more naïve vaccinees, especially those whose vaccine-elicited titers reach only Q3, Q2 or Q1. (I am not asking for additional data [unless it happens to exist] but feel that this limitation of the study deserves more airplay in the Discussion)."

Response: We thank the reviewer for this important observation. Unfortunately, under our ethical obligations, our only accesses to naïve vaccinated individuals were from institutional employees who agreed to volunteer for this study. For this reason, our resources were limited and thus we reached a pool of only 17 individuals. Nonetheless, as the reviewer suggested, it is important to note this limitation. We have added a note in the discussion section referring to this limitation (line 338-341) as well as in the limitation of study section (lines 370-375).

**Reviewer's comment:** “The fact that this study lacks analysis of antibody responses directed to the spike ectodomain, and has focused exclusively on the RBD, should be discussed. For instance, the antibody decay rate of S-directed titers between recovered patient's vs naïve vaccinees might be statistically indistinguishable, thus diminishing the key observation manuscript in the current manuscript (namely, a difference in antibody persistence of RBD-directed IgG between these two groups)."

**Response:** We thank the reviewer for this insightful note. Indeed, we agree that it is important to clarify the reasoning for the exclusive focus on RBD, rather than the whole spike. We have taken the reviewers comment into consideration and added and additional elaboration on this point, which can be found in the “Introduction” section, lines 66-79.

In addition, we acknowledge the reviewer's concern for the effectiveness of the antibody decay rate of S-directed titers between recovered patient's vs naïve vaccinees. In the revised manuscript we discuss this limitation as well (lines 376-382).

## Part III

**Reviewer's comment:** “There is no callout of Fig. 3c in the caption."

**Response:** We thank the reviewer for this note and apologize for the mistake. Indeed, an error was made in Fig 3c), and in general the figures in the revised manuscript have been improved. The required addition can be found in the legend of Fig 5 (formerly Figure 3c).

**Reviewer's comment:** “It is unclear how exactly the statistical tests were applied in Fig. 3c"

**Response:** We thank the reviewer for this request for clarification on the statistical comparison made in this instance. In Fig 5 (formerly Figure 3c), we present to summary of the results of multiple comparison of the regression coefficients as determined for each isotype (by applying GAMM analysis. We have revised an expanded clarification on this point in the manuscript,

so that this comparison might be clear to readers from all disciplines. This clarification can be found in the legend of Fig 5.

**Reviewer's comment:** “The first paragraph of the Discussion is dispensable and could be deleted."

**Response:** We agree and have deleted that text.
