# Supplementary material for: Longitudinal kinetics of RBD+ antibodies in COVID-19 recovered patients over 14 months
Source: PLoS Pathog. 2022 Jun 3;18(6):e1010569. doi: 10.1371/journal.ppat.1010569 (PMC9200310; doi:10.1371/journal.ppat.1010569)
Supplement: Attachment — Submitted filename: Response to reviewers.pdf [file ppat.1010569.s013.pdf]

Monday, 11 April 2022

Dear PLOS Pathogens editors,

We would like to begin by thanking reviewers for highlighting the improvement made in the revised manuscript entitled; "**Longitudinal kinetics of RBD$^+$ antibodies in COVID-19 recovered patients over 14 months**" manuscript number, PPATHOGENS-D-21-02063.

Both reviewer#1 and reviewer#3 stated that all their concerns were fully addressed. We thank again all reviewers for their constructive inputs.

We have carefully considered and addressed reviewer#2 important comment and revised the manuscript according to his/her suggestion.

The detailed response to the reviewers' comments can be found below.

We thank you for the consideration of our work and look forward to hearing from you.

Sincerely,

Yariv Wine, PhD

Part I - Summary

Reviewer #2: Overall, the authors have responded to my concerns. However, they failed to respond to a particular concern about bias and their response is concerning.

Response to reviewer: We apologize for not addressing the particular concern about bias and believe our response will be satisfactory (see part II).

Part II – Major Issues: Key Experiments Required for Acceptance

Reviewer #2: The comment and response in question is:
Reviewer's comment: "At a minimum basic information on the characteristics (age, sex, severity) of individuals that contributed at each time point should be presented along with test statistics to evaluate differences.

" Response: The reviewer's request for an addition of presented information on the characteristics (age, sex, severity) of individuals who contributed at each time point is very much understandable. However, we found this kind of presentation sub-optimal and disproportional to the facts. This is the sole reason that while aiming for a 3-month interval between blood sampling, in practice, we had no executive control of the matter (participants would arrive on their own accord). This results in a non-uniform sampling time frame between participants.

While I completely understand that non-uniform sampling time occurs in observational studies. The authors in question have binned the sampling times and thus there is no reason that the information on who contributed samples at which time points cannot be presented at least in the supplement. They should just bin using the same time points. Their reluctance to provide basic data on their samples and the minimum data necessary to evaluate potential bias in this study due to loss-to-follow up is very concerning.

Response to reviewer: We completely understand the concern raised by the reviewer and apologize for not providing the information during the first revision due to misunderstanding the original comment. We were under the impression that the reviewer requested information at the individual level that would have compromised the privacy of our donors. The sole reason for excluding this information was for protecting the donor's privacy in accordance with the ethical guidelines. Following the comment in the second revision, the reviewer requested information regarding age, sex and severity in accordance with the binned sampling times (V1-V4). Thus, we have now included as supplement Figure 1 the requested information. We believe that this information will address the major concern raised by the reviewer.
